# Skeletal and dental tissue mineralization: The potential role of the endoplasmic reticulum/Golgi complex and the endolysosomal and autophagic transport systems

**DOI:** 10.1016/j.bone.2025.117390

**Published:** 2025-01-13

**Authors:** Irving M. Shapiro, Makarand V. Risbud, Tengteng Tang, William J. Landis

**Affiliations:** a Department of Orthopaedic Surgery, Sidney Kimmel Medical College, Thomas Jefferson University, Philadelphia, PA, United States of America; b Center for Applied Biomechanics, Department of Mechanical and Aerospace Engineering, School of Engineering and Applied Science, University of Virginia, Charlottesville, VA, United States of America; c Department of Preventive and Restorative Dental Sciences, School of Dentistry, University of California at San Francisco, San Francisco, CA, United States of America

**Keywords:** Endoplasmic reticulum/Golgi complex, Mitochondria, Calcium and phosphate ions, Endolysosomal transport system, Autophagic transport system, Matrix vesicle, Mineralization

## Abstract

This paper presents a review of the potential role of the endoplasmic reticulum/Golgi complex and intracellular vesicles in mediating events leading to or associated with vertebrate tissue mineralization. The possible importance of these organelles in this process is suggested by observations that calcium ions accumulate in the tubules and lacunae of the endoplasmic reticulum and Golgi. Similar levels of calcium ions (approaching millimolar) are present in vesicles derived from endosomes, lysosomes and autophagosomes. The cellular level of phosphate ions in these organelles is also high (millimolar). While the source of these ions for mineral formation has not been identified, there are sound reasons for considering that they may be liberated from mitochondria during the utilization of ATP for anabolic purposes, perhaps linked to matrix synthesis. Published studies indicate that calcium and phosphate ions or their clusters contained as cargo within the intracellular organelles noted above lead to formation of extracellular mineral. The mineral sequestered in mitochondria has been documented as an amorphous calcium phosphate. The ion-, ion cluster- or mineral-containing vesicles exit the cell in plasma membrane blebs, secretory lysosomes or possibly intraluminal vesicles. Such a cell-regulated process provides a means for the rapid transport of ions or mineral particles to the mineralization front of skeletal and dental tissues. Within the extracellular matrix, the ions or mineral may associate to form larger aggregates and potential mineral nuclei, and they may bind to collagen and other proteins. How cells of hard tissues perform their housekeeping and other biosynthetic functions while transporting the very large volumes of ions required for mineralization of the extracellular matrix is far from clear. Addressing this and related questions raised in this review suggests guidelines for further investigations of the intracellular processes promoting the mineralization of the skeletal and dental tissues.

## 1. Introduction: an overview of the involvement of the endoplasmic reticulum/Golgi complex with calcium ion homeostasis and mineralization

With the exception of dental enamel, current understanding of the mechanism of vertebrate mineralization is based principally on the unique properties of the extracellular protein, collagen, and membrane-bound organelles, matrix vesicles. Recent technological developments that engage cells undergoing mineralization, together with more complete knowledge of calcium ion homeostasis, have generated new insight into the role of cells and cell-derived vesicles, including matrix vesicles, in mineral deposition. In the light of these advancements, mineralization may now be considered in terms of intracellular events supportive of extracellular activities, and the former are centered about the actions of the endoplasmic reticulum/Golgi complex in regulating mineral deposition. Briefly, calcium ions are initially accumulated in the endoplasmic reticulum and subsequently transported in a variety of vesicles to the plasma membrane. Following release from the cell, the trafficked calcium ions, along with phosphate ions originating from the same vesicles or other cellular sources, contribute to the formation, growth and maturation of the mineral, hydroxyapatite. The mineralization process is directed by the protein architecture of the extracellular matrix, penetrating vascular elements, biomechanical forces and additional factors, resulting in the deposition in the adult human male skeleton of about 9 kg of apatitic mineral, comprising approximately 12% of body weight [1].

**Fig. 1.**
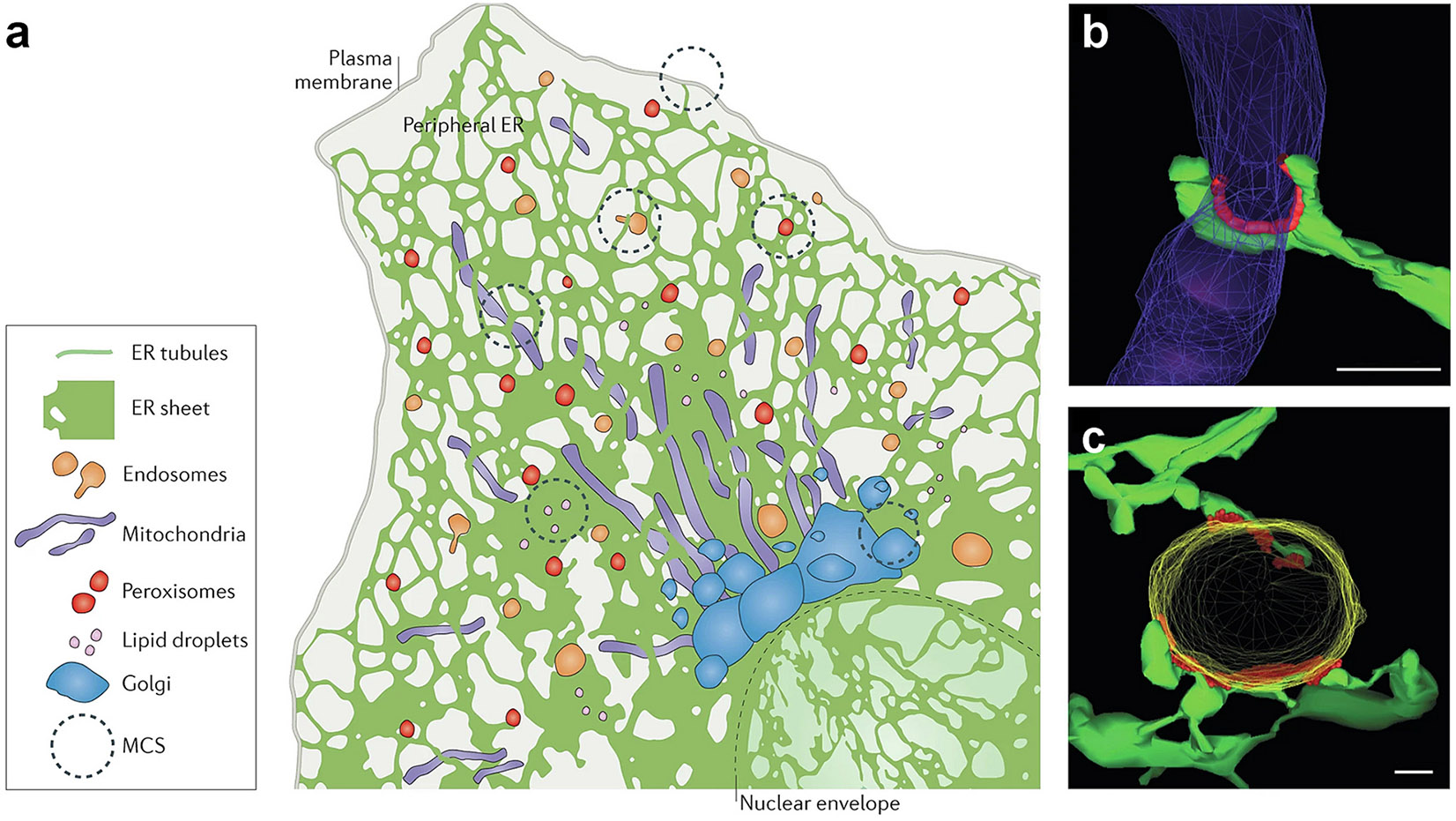
Structural interaction of the endoplasmic reticulum with the plasma membrane and other organelles of a eukaryotic cell. (a) The endoplasmic reticulum (green) is the largest membranous component in a cell and it associates with mitochondria (purple), the Golgi apparatus (blue), and endosomes (orange) as well as peroxisomes and lipid droplets. Tubules of the endoplasmic reticulum form dynamic contacts with sites on the plasma membrane (MCS). Much of the endoplasmic reticulum is located around the nucleus (defined by the nuclear membrane), and its tubules and sheets extend outward and throughout the cytoplasm to contact the organelles noted above. (b, c) Image reconstructions from electron tomography reveal contact between tubules of the endoplasmic reticulum (green) and (b) the plasma membrane of a yeast cell (red) and (c) an endosome of an animal cell (yellow). Scale bar = 200 nm for both (b) and (c). Images from Phillips and Voeltz [6] with permission.

To precede a discussion of how the endoplasmic reticulum is involved with vertebrate tissue mineralization, it is interesting to note the discovery and function of this organelle. Toward the end of the 19th century, two major findings profoundly influenced the understanding of the microstructure and function of the cell. In 1897, the French scientist, Charles Garnier, studying the structure of glandular tissue, described a new constituent that he called the Ergastoplasm [2]. This organelle, renamed the endoplasmic reticulum (reticulum = network), was the largest component in the cell and of considerable complexity. A year later, Camillo Golgi chronicled a novel cellular structure, the “*apparato reticolare interno,*” in the nerves of the barn owl [3]. The new organelle was subsequently known as the Golgi complex or apparatus. With the advent of the electron microscope, it was found that the endoplasmic reticulum and the Golgi apparatus were closely associated. Microscopic images also revealed the intricacy of both organelles: the Golgi apparatus exhibited a flattened membranous cisterna, arranged like a mound of pancakes. At a distance from the nucleus, the endoplasmic reticulum was comprised of helicoidal layers of membranous sheets forming a meshwork of densely packed tubular channels with stacked flat cisternae (Fig. 1) [4-6]. It was thought that the lumen of the tubular channels and cisternae were continuous with each other throughout the endoplasmic reticulum network and that the type of morphology might be tissue-specific [7].

**Fig. 2.**
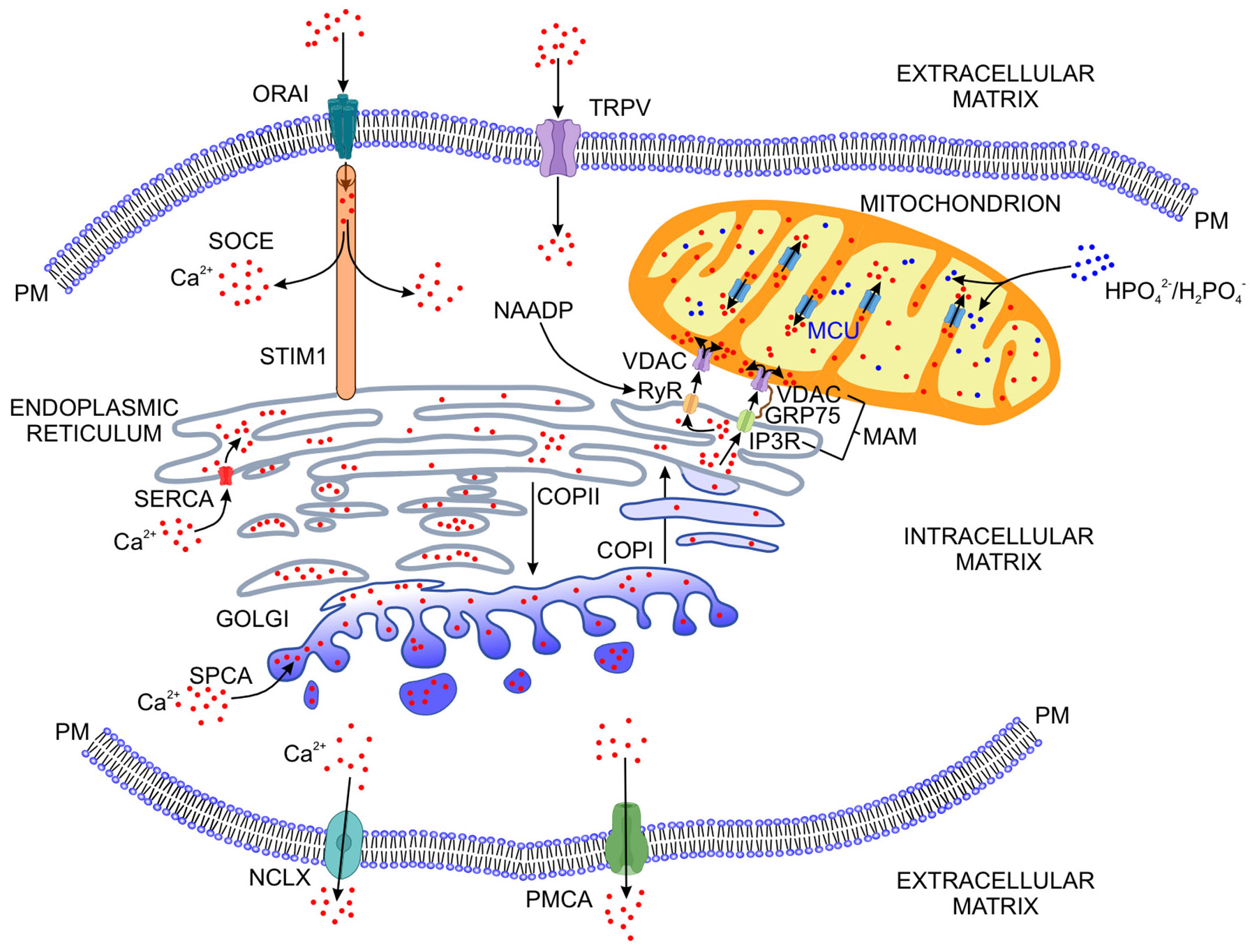
Schematic of calcium ion homeostasis in cells of vertebrate mineralizing tissues. Several ligand-gated and voltage-gated channels in the plasma membrane (PM) serve to maintain the ionized calcium level of the intracellular matrix (cytosol). Non-selective ion channels including TRPV/TRPM [29-31] and ORAI-STIM [40-44] are among the channels most active in facilitating calcium ion (Ca^2+^, red circles) transport into the cytosol while the concerted activities of plasma membrane PMCA and NCLX channels regulate calcium ion exit from the cell [27,28,35]. Not shown are details of voltage-gated calcium ion channels as they are largely excluded from non-excitable cells. Likewise, receptor-operated channels, which open rapidly upon binding an external ligand, are also preponderate in excitable cells. Along with the plasma membrane channel proteins, the endoplasmic reticulum (ER)-Golgi complex and mitochondria play key intracellular roles in calcium ion homeostasis. For example, responding to an increase in cytosolic calcium ion levels, a sarcoendoplasmic reticulum ATPase (SERCA) pumps the cation into the endoplasmic reticulum [45,48]. Additionally, secretory pathway calcium ATPase (SPCA) shuttles calcium ions from the cytosol into the Golgi [51,52]. The stromal interaction molecule (STIM) senses and controls the ER calcium ion content [40,43,44]. When this activity decreases, STIM oligomerizes with the plasma membrane calcium channel protein, ORAI1, to open a channel for entry of calcium ions into the cytosol [39,41,42]. This refilling activity, store operated calcium entry (SOCE), appears to be widespread and likely represents the major calcium entry pathway into the cell [31,39]. Calcium and other ions along with proteins accumulated in the ER are trafficked to the *cis* end of the Golgi (light blue) in stacks of vesicles that bud from ER membranes [9,10]. Vesicle budding and movement from ER to Golgi are facilitated by COPII (a coatamer) that coats the vesicles with a specific protein complex utilized for this anterograde membrane transport [9]. COPI, another coatamer, mediates budding and retrograde transport of vesicles with spent or defective cargo from the *cis*-Golgi to the ER [14]. Further control of the cytosolic calcium ion level is mediated by physical and functional coupling of the endoplasmic reticulum to mitochondria by a protein platform, the mitochondria-associated endoplasmic reticulum membrane (MAM) [65,67]. Major MAM proteins include GRP-75, VDAC1 and IP3R. The function of the MAM is to promote and regulate calcium ion release from the endoplasmic reticulum by the water-soluble phospholipid, IP3. This ligand is produced from the membrane phospholipid by the activity of phospholipase C. When IP3 is bound to its cognate receptor (IP3R) in the endoplasmic reticulum, calcium ions are liberated from the endoplasmic reticulum stores to mitochondria and the cytosol. Another family of endoplasmic reticulum resident channel proteins (ryanodine receptors, RyR) is sensitive to the NAD metabolite, nicotinic acid adenine dinucleotide phosphate (NAADP). When NAADP binds to RyR, calcium ions are again released from the endoplasmic reticulum to mitochondria. The schematic additionally shows entry of cytosolic phosphate ions (${\text{HPO}}_{4}^{2-}∕H_{2}{\text{PO}}_{4}^{-}$, blue circles) into a mitochondrion, where they contribute to regulation of calcium ion homeostasis through the formation of calcium phosphate ion clusters in these organelles. The *trans*-end of the Golgi (dark blue) gives rise to a variety of intracellular vesicles, including endosomes and lysosomes. (Figure created using BioRender.com.)

The functional relationship between the endoplasmic reticulum and Golgi apparatus is now known to be very intimate: The ribosomes and polyribosomes that decorate the cytoplasmic faces of the endoplasmic reticulum provide surfaces for ATP-dependent polypeptide chain synthesis. The proteins are modified, sorted and incorporated into transport vesicles coated with a protein complex, COPII, a coatamer (Fig. 2) [8-10]. These vesicles bud off from ribosome-free areas of the endoplasmic reticulum and traffic in an anterograde manner to the Golgi complex [8,11]. Within the Golgi, peptides and lipid molecules are attached to the nascent proteins. Once correctly folded and assembled, many of the proteins are targeted to the plasma membrane for secretion, while a different set of transport vesicles, coated with another coatamer, COPI, originating with the Golgi, returns unfolded or misaligned proteins to the endoplasmic reticulum for further processing (retrograde trafficking) (Fig. 2) [9,10,12-14]. Besides peptides and lipids, the endoplasmic reticulum/Golgi apparatus contains high levels of calcium ions, which are also packaged into the coated vesicles [15]. Protein complexes called translocons, specifically the Sec61 translocon, function to minimize the loss of these ions during nascent peptide transit [16]. Additionally, the endoplasmic reticulum/Golgi apparatus gives rise to other vesicles which transport their cargo, including calcium ions, along various pathways and throughout the cell. Some of these vesicles comprise organelles of the endolysosomal and autophagic systems of a cell and are related to the mineralization process in vertebrate hard tissues, as will be discussed below.

From these initial remarks, then, and relevant to the central theme of this review, the endoplasmic reticulum together with the Golgi complex regulates calcium ion homeostasis and generates many of the transport vesicles involved with mineral deposition [17]. In this context, the paper addresses the mechanism of cellular ion loading and uptake by the endoplasmic reticulum/Golgi complex as well as several other aspects of the cellular regulation of mineral deposition. These include the possible role of mitochondria as a source of phosphate ions for mineral formation, strategies employed by the cell to traffic calcium ions from storage organelles to the plasma membrane, and processes that sort calcium-loaded vesicles from other organelles. Finally, the paper discusses mineral transport in vesicles originating from the endoplasmic reticulum/Golgi system and the role of extracellular matrix vesicles in mediating the mineralization of bone, dentin, cartilage and other vertebrate hard tissues. For convenience in this review and the fact that the tubules and lacunae of the endoplasmic reticulum are present throughout the cytoplasm, the use of the term, “endoplasmic reticulum,” is inclusive of the Golgi complex.

## 2. Mechanism of cellular ion loading and uptake by the endoplasmic reticulum

**Fig. 3.**
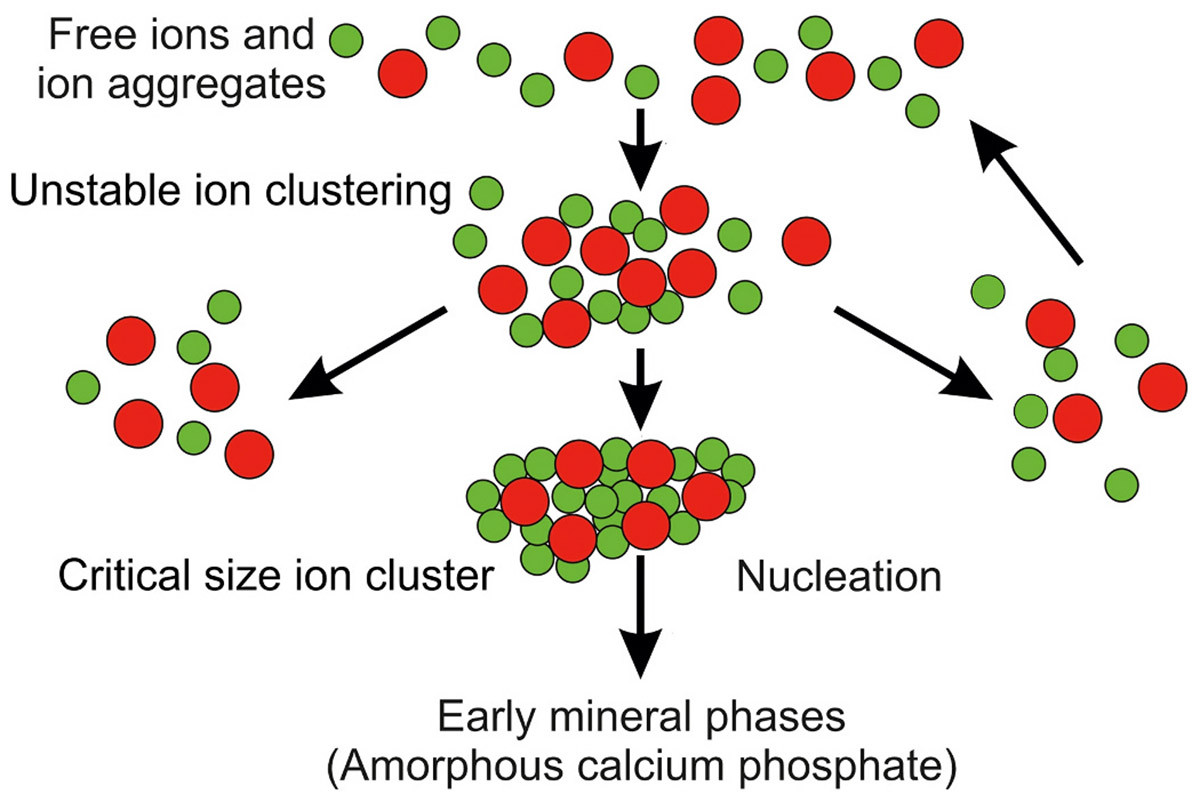
Diagram illustrating mineral ions and their possible interactions within intracellular vesicles originating from the endoplasmic reticulum/Golgi complex of a cell. Calcium (green) and phosphate (red) ions accumulated and sequestered in the vesicles may undergo changes in free energy leading to the formation of ion clusters. The first clusters formed are relatively unstable because of destabilizing effects of high surface energy, and the ions comprising such clusters may dissociate and become non-aggregated once again. When the rate of ion accumulation exceeds the rate of ion liberation into the bulk vesicle fluid, stable ion clusters of critical size (embryos or nuclei) of calcium phosphate form. Changes in free energy of the system may enhance additional ion accumulation and an initial rudimentary ordering or organization of the ions into an early mineral phase occurs. In the case of bone, such an early phase may be an amorphous calcium phosphate whose ions possess only short-range order. Subsequent events within the vesicles may lead to transformation of amorphous calcium phosphate into other mineral phases such as brushite or octacalcium phosphate. (Figure adapted with permission from Shapiro and Landis [26].)

The endoplasmic reticulum provides a cellular store of calcium ions that may be utilized for the mineralization process (Fig. 2). This role is predicated in part by plasma membrane control of the cytosolic calcium ion concentration. To participate directly in the deposition of mineral in a developing vertebrate tissue, a cell is required to process and export large quantities of calcium and phosphate ions. For example, in the final weeks of pregnancy for a human embryo, over 300 mg of calcium and 200 mg of phosphorus are taken up daily by mineralizing bone [18]. How the osteoblast meets these ion levels in the developing fetus is poorly understood, especially as the cytosolic calcium level is very low (~100 nM) and at first glance insufficient to support the kinetics of mineral formation in vitro or in vivo. According to Laude and Simpson, the cytosolic calcium value is 10,000-20,000-fold lower than that of the extracellular tissue fluid (1.0–2.0 mM) [19]. Hence, the release of ions from the cytosol would add little to the extracellular calcium ion concentration and would be inadequate to promote intra- or extracellular mineral deposition. However, as will be discussed shortly, the extensive cisternae and tubules of the endoplasmic reticulum accumulate high concentrations of calcium and phosphate ions, such that the actual calcium ion concentration can vary between 400 and 800 μM [19]. Like the endoplasmic reticulum, the Golgi complex also stores elevated concentrations of calcium ions, which can be mobilized with ligands binding inositol 1,4,5-trisphosphate (IP3R) and ryanodine receptors (RyR) [20,21]. Contained in small-volume vesicles that bud off from these organelles, calcium ions, together with phosphate ions, would be expected to associate with one another and possibly form pre-nucleation ion clusters [22,23]. Within the crowded environment of these vesicles, a change in the Gibbs free energy would favor transitions of calcium and phosphate ion clusters to more energetically stable mineral species, such as amorphous calcium phosphate, apatite or intermediates between these two mineral phases (Fig. 3) [24-26]. Moreover, a very active vesicular transport system could rapidly traffic these examples of ions, ion clusters or putative preformed mineral cargo to the extracellular matrix. In this way, the many metabolically active osteoblasts that line an osteoid seam, for instance, can transport the quantities of mineral needed to initiate or drive mineralization of the extracellular matrix of a developing bone. Growth and development of the various mineral species in the ion-rich extracellular tissue fluid would ultimately form the final apatitic phase of skeletal and dental tissues.

As a first step toward understanding how the endoplasmic reticulum loads with calcium ions, it is important to recognize that the plasma membrane creates an almost impenetrable barrier against accidental or possibly uncontrolled leakage of ions into the cytosol. This membrane, however, maintains the activity of many calcium-dependent molecular circuits by exquisitely regulating entry of mono- and divalent ions into the cell through voltage-gated and receptor-operated calcium channels located in its lipid membrane bilayer (Fig. 2). Fine control of cytosolic calcium homeostasis is supported by a plasma membrane calcium ATPase (PMCA) and a sodium/calcium/lithium exchanger (NCLX) (Fig. 2) [27,28]. The exchanger, NCLX, is thought to counteract any large cytosolic calcium fluctuations while PMCA maintains cytosolic calcium homeostasis [27]. Another series of plasma membrane channels, transient receptor potential (TRP) melastatin (TRPM), forms membrane pores that are permeable to divalent cations and regulates magnesium and calcium ion homeostasis [29]. As will be discussed later in this review, these TRPM proteins are also localized in the endoplasmic reticulum [30], where they are associated with membrane calcium-selective calcium release-activated calcium (CRAC) channels [31]. Such CRAC channels mediate store-operated calcium entry (SOCE) into cells: SOCE responds to a low calcium ion content of the endoplasmic reticulum by refilling the cytosol with calcium ions from the extracellular tissue fluid (Fig. 2) [32].

Not unexpectedly, the protein pump systems noted above control and maintain cytosolic calcium homeostasis in the cells of a variety of vertebrate hard tissues. For example, TRPM7 is expressed in osteoblasts and growth plate chondrocytes [29,33,34]. Deletion of the *TRPM7* gene impairs bone growth in the former cells [33] and leads to downregulation of expression of *type X collagen, Indian hedgehog* and *matrix metalloproteinase-13* in hypertrophic chondrocytes of growth cartilage [34]. In cells of enamel, dentin and craniofacial bone, a heterozygous mutation of the TRPM7 kinase domain causes a severe hypomineralization phenotype [29]. Since magnesium ions corrected the mineralization defect, Nakano et al. concluded that the kinase provided magnesium ions for optimum function of tissue non-specific alkaline phosphatase (TNAP) in ameloblasts, odontoblasts and osteoblasts [29]. In this regard, it is likely that *TRPM7* is required for regulating the cellular levels of both calcium and magnesium ions and thereby plays a critical role in events directly linked to the vertebrate mineralization process.

NCLX and NCX pumps are present in the plasma membranes of both soft and hard tissues and serve to drive calcium ions into the extracellular environment from the cytosol. (Fig. 2) Stains, Weber and Gay showed the NCX3 isoform was expressed by osteoblasts and localized to the osteoblast membrane adjacent to osteoid [35]. Based on its expression pattern and spatial distribution, they suggested that NCX was present in osteoblasts and activated during the initial stages of bone mineralization [35]. During ion loading of the endoplasmic reticulum and Golgi apparatus, it is likely that activation of the pumps mentioned above maintains cell function by preventing calcium ions from causing senescence and apoptosis. Results of each of these investigations indicate the importance of TRPM7 and NCLX in bone and tooth mineralization. The central role of SOCE and CRAC channels in regulating calcium ion uptake by cells is presented below.

In addition to the control exerted by the plasma membrane, calcium ion loading of the endoplasmic reticulum depends as well on SOCE (Fig. 2). As noted earlier, the observation that deletion of the *TRPM* gene exerted a partial effect on mineralization of several different vertebrate tissues implies that other channel proteins may play a critical role in regulating calcium ion entry into cells. Relevant to this consideration, Faouzi and co-workers confirmed that, besides serving as a channel protein, TRPM7 controlled the activity of the plasma membrane channel protein, ORAI (Fig. 2) [36]. This protein functions as a component of the CRAC channel [37,38]. Cioffi et al. proposed that the CRAC channel was a ternary complex formed by ORAI1, TRPcanonical(C)1 and the evolutionarily conserved stromal interaction molecule (STIM) (Fig. 2) [39].

SOCE is activated when the calcium ion level in the lacunae of the endoplasmic reticulum falls, an event that induces a refilling response of these ions [40]. The decrease in the calcium ion level is sensed by membrane-anchored STIM proteins in the endoplasmic reticulum [40]. As calcium ions dissociate from EF-hand motifs of STIM, the protein oligomerizes and binds the cytosolic region of ORAI [40]. A protein-protein complex is then formed connecting the plasma membrane with the membranes of the endoplasmic reticulum, and a pore, orientated perpendicular to the plasma membrane, is created in the ORAI protein that permits calcium ions to enter the cytosol from the extracellular tissue fluid (Fig. 2) [40-42] Calcium ion entry into the cell in this manner is facilitated by the existence of the very large ion gradient across the plasma membrane, as mentioned above.

The STIM isoforms, STIM1 and STIM2, vary in their ability to sense endoplasmic reticulum calcium ion levels: STIM1 senses levels below 400 μM and activates SOCE; STIM2 senses smaller changes in the basal ion level and thus has been associated with calcium ion homeostasis in the endoplasmic reticulum [43]. Once the endoplasmic reticulum refills with cytosolic calcium ions, the connection between STIM and ORAI is severed. The calcium ions disrupt the association between the STIM oligomers of the endoplasmic reticulum. As a result, interactions with membrane ORAI proteins are reversed and the CRAC ion channel closes [44].

It should be emphasized that although the SOCE system provides a regulated inflow of calcium ions into the cell, the ions are released into the cytosol and not into the endoplasmic reticulum (Fig. 2). Instead, the increase in cytosolic calcium ion levels provokes the activities of cellular systems that function to maintain ion homeostasis. As noted earlier, these systems are active at the level of the plasma membrane, mitochondria and the endoplasmic reticulum. A (sarco)endoplasmic reticulum calcium ATPase (SERCA) pumps calcium ions from the cytosol into the lumen of the endoplasmic reticulum (Fig. 2) [19,45]. The basis for this action is the promotion of cation uptake by the reduced state of SERCA thiol cysteine residues [46,47]. As its name suggests, SERCA uses the energy released from ATP to transport two calcium ions from the cytosol into the endoplasmic reticulum lumen in exchange for two protons exported from the lumen into the cytosol. This activity is regulated by two membrane proteins, phospholamban and sarcolipin [48]. When the endoplasmic reticulum calcium ion level is maximal (high micromolar range, ~100 μM), the luminal chaperones, calnexin and calreticulin, limit further calcium uptake [49]. Restoration of the luminal calcium ion levels is associated with oxidation of the SERCA cysteine residues and ion import arrest [50]. Aside from SERCA, a secretory pathway calcium ATPase (SPCA) moves cytosolic calcium ions into the Golgi from the cytosol (Fig. 2) [51,52].

The importance of SOCE function in relation to mineralization has been documented in numerous studies. Both *ORAI* and *STIM* transcripts are expressed in bone [53]. Overexpression of *STIM1* enhances osteoblastic differentiation and promotes mineral deposition and the secretion of selected osteogenic markers, Runx2, type I collagen, and bone morphogenetic protein 4 [54]. Deletion of *STIM1* in mice produces a phenotype with marked skeletal abnormalities that include a reduced number of ribs and kyphosis of the spine [55]. The bone architecture in these animals is abnormal with increased trabecular and decreased cortical bone [55]. Disruption of expression of *ORAI1* inhibits osteoblast differentiation and delays skeletal development [56,57]. Moreover, deletion of *ORAI1* reduces SOCE activity and prevents the formation of mineralizing nodules [58]. On the other hand, when ORAI1 channel activity is upregulated, there is enhanced SOCE activity [59].

In dental tissues, STIM is required for tooth development and mineralization. Both STIM1 and ORAI1 proteins are present in ameloblasts and localized to the endoplasmic reticulum and plasma membrane, respectively [60]. The phenotype of *STIM1* knockout mice includes a reduction in dentin thickness and mineral density and an alveolar bone that is hypomineralized with changes in trabecular number, trabecular thickness and total bone volume [61]. A dental phenotype with chalky enamel is also observed in *STIM1*-deficient mice [55,62]. In addition to defects in human enamel formation, there is “excessive enamel wear, or attrition or loss, related to poor mineralization, and discoloration of the residual enamel, a condition observed in both deciduous and permanent teeth.” [63] Results of these studies of dental and skeletal tissues indicate that, in concert with those of soft tissues, SOCE is an active calcium homeostatic system and intimately concerned with regulation of the mineralization response.

Of note, while calcium ions play multiple roles in the cell, if calcium homeostasis is not maintained, then apoptosis ensues. From this perspective, the increase in cytosolic calcium ions during SOCE is maintained by the coordinated activities of the endoplasmic reticulum and mitochondria. Mitochondria take up calcium ions from the cytosol [64] and receive calcium ions directly from the endoplasmic reticulum by spatially restricted mitochondria-associated endoplasmic reticulum membrane (MAM) proteins (Fig. 2). The MAM molecular structure is complex: Inositol 3 phosphate receptor proteins, (IP3R), located in the endoplasmic reticulum membrane, associate with the voltage-dependent anion channel protein (VDAC)1 in the outer mitochondrial membrane [65]. These proteins complex with the mitochondrial inner membrane calcium uniporter (MCU), stabilized by a cytosolic 75 kDa glucose-regulated protein (GRP75) (Fig. 2) [66]. When IP3 is bound to its receptor (IP3R), a conformational change occurs in the MCU protein that enables the MAM platform to transfer calcium ions from the endoplasmic reticulum into the mitochondrial matrix (Fig. 2) [67]. Calcium ion transit between the organelles is enhanced by the large negative membrane potential across the inner mitochondrial membrane. In this way, in its association with mitochondria, the endoplasmic reticulum can load with levels of calcium ions required for optimum function while low calcium ion levels are maintained in the cytosol. As will be discussed shortly, vesicles containing calcium ions may be derived from the endoplasmic reticulum to participate in subsequent intracellular events.

In addition to MAM complexes playing a key role in inter-organelle calcium transport and homeostasis, other complexes such as AXER (ATP/ADP exchanger in the endoplasmic reticulum membrane) [68,69], facilitate ATP movement from the mitochondrion to the endoplasmic reticulum [70]. While a discussion of AXER and ATP transport and utilization may seem to be unrelated to apatite formation, there is the likelihood that the metabolic intermediate ATP may serve as a source of phosphate ions for the mineralization process, as noted previously. Indeed, kilograms of phosphate ions are released daily from ATP by the numerous ATP-utilizing systems that populate the endoplasmic reticulum membrane [71]. For example, during protein synthesis, aminoacyl-tRNA synthetase utilizes 2 ATP molecules to catalyze the esterification of an amino acid to its cognate tRNA; the subsequent elongation and translocation steps utilize 2 GTPs. The reaction is driven by the free energy change (ΔG – 33 kJ/mol, pH 7.0) associated with the hydrolysis of pyrophosphate (PPi) to inorganic phosphate (Pi):

Amino acid+2ATP→AminoacylAMP+2PPi2PPi→4Pi

Of course, there is the possibility that residual pyrophosphate may stabilize ion clusters or even apatitic mineral [72]. Likewise, the hydrolysis of ATP or the accumulation of phosphate ions may promote vesicle blebbing of the plasma membrane [73]. However, as will be discussed later, it is known that vesicles derived from the endoplasmic reticulum, such as endolysosomes and autophagosomes, accumulate ions and deliver mineral particles to the plasma membrane.

To accommodate the elevated energy needs of the endoplasmic reticulum during new bone formation, for example, or the mineralization of other vertebrate hard tissues, it would not be unreasonable to assume that the rate of ATP synthesis would be elevated. In the hypoxic environment of forming bone, hypoxia inducible factor (HIF)-1α would both upregulate glycolytic activity and promote the expression and secretion of hypoxia upregulated mitochondrial movement regulator (HUMMR) [74]. This protein mediates the distribution and transport of mitochondria and facilitates the association of mitochondria with the endoplasmic reticulum [75]. The mitochondrial movements and interactions would likely enhance delivery of ATP and phosphate ions to the endoplasmic reticulum. Curiously, the transport of ATP from mitochondria to the endoplasmic reticulum is influenced by the cytosolic calcium ion concentration. Thus, Yong and co-workers reported that a rise in the cytosolic calcium ion concentration inhibited ATP import into the lumen of the endoplasmic reticulum [76]. In other words, ATP availability is increased as calcium ions are removed from the cytosol by the endoplasmic reticulum and mitochondria.

Regarding ATP, its synthesis is dependent on several factors, of which the most important are the transport and availability of the phosphate anion. In an earlier review of the types of transporters that support phosphate ion entry into the cells of skeletal and dental tissues, Shapiro and Landis [26] drew attention to work that indicated the importance of the phosphate transporter proteins, SLC20A1/PiT1, SLC20A2/PiT2 and SLC34A1/NaPi-IIa. It was noted that deletion of SLC20A2/PiT2 caused disorders of skeletal and dental tissues, conditional deletion of SLC20A1/PiT1 impaired endochondral ossification, and phosphate transporter loss led to endoplasmic reticulum stress with retention of aggrecan and occasional chondrocyte death [26]. Additionally, ligand binding of inositol hexakisphosphate (IP6) to XPR1 (xenotropic and polytropic murine leukemia retrovirus 1 multipass membrane receptor) promoted phosphate ion liberation from cells while loss of IP6 resulted in increased ATP and intracellular free phosphate together with decreased cellular release of phosphate ions [26]. Stated in another way, the utilization of ATP for biosynthetic activities of the endoplasmic reticulum results in the liberation of large quantities of phosphate ions from cells.

In summary, utilization of ATP for biosynthetic activities of the endoplasmic reticulum results in release of large quantities of phosphate ions. Although the fate of these ions is unknown, it is plausible that they may be incorporated into the network of tubules and lacunae of the endoplasmic reticulum or into vesicles associated with the organelle that are subsequently destined for transport to the extracellular matrix. From the perspective of the mineralization process, ATP may thereby provide phosphate ions for mineral formation. Of course, the presence of increased concentrations of calcium and phosphate ions, whether associated with intracellular organelles or free in the cytosol, would undoubtedly influence the numerous molecular pathways and circuits that control cell function. In the case of osteoblasts or other vertebrate hard tissue cells, it is likely that regulatory systems have evolved to accommodate metabolic changes associated with calcium and phosphate ion release, storage and transit. These system modifications might include changes in IP3R and RyR density and STIM-calcium affinity, the capacity of the endoplasmic reticulum to store calcium, the influence of luminal calcium-binding proteins in the endoplasmic reticulum, and the specificity of interactions between the endoplasmic reticulum and mitochondria. How the influence of these modifications on vesicle calcium and phosphate ion sequestration, accumulation, clustering and transit from the endoplasmic reticulum to and away from the plasma membrane is considered below. Discussion will center on the liberation of calcium ions from the endoplasmic reticulum and the identification of vesicles transporting them, the means of sorting and trafficking cargo-laden vesicles, the interaction of loaded vesicles with the plasma membrane, and the export of mineral ions or ion-containing vesicles from the plasma membrane to the extracellular milieu.

## 3. Release of calcium stores from the endoplasmic reticulum and uptake by transport vesicles

**Fig. 4.**
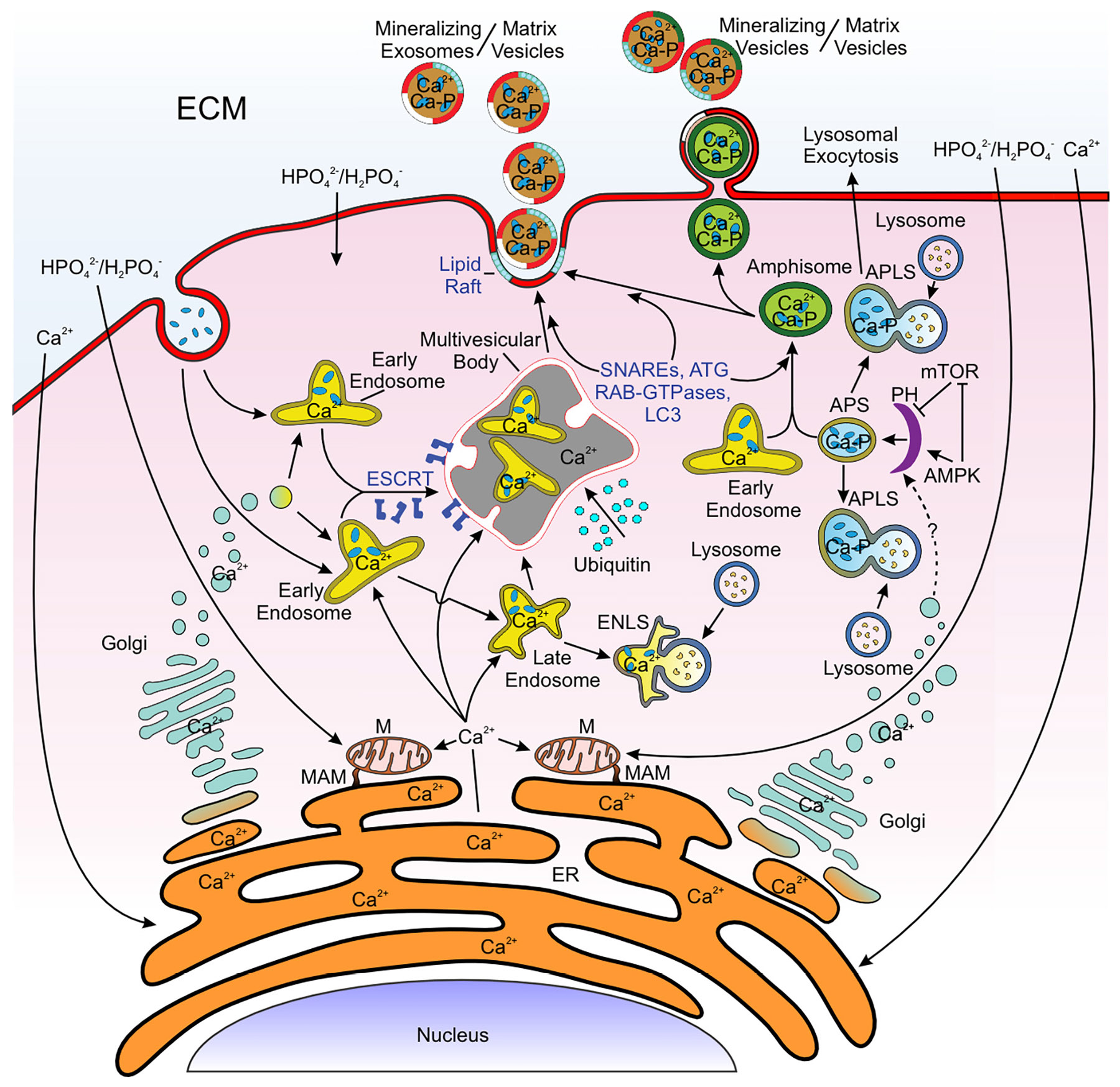
Schematic of intracellular vesicles trafficking cargo from the endoplasmic reticulum to the plasma membrane and extracellular matrix. The endoplasmic reticulum (ER) and the Golgi apparatus (G) form tubular networks that contain high concentrations of calcium ions (Ca^2+^). These ions are packaged as cargo in small blebs or stacks of vesicles that bud from the ER and G and are trafficked between the two organelles bidirectionally. This type of anterograde - retrograde transport is mediated by specific protein complexes (COPI, COPII) that coat the transport vesicles as shown in Fig. 2. Budding from the ER/G complex are additional distinct groups of vesicles, lysosomes and endosomes, which traffic calcium ions and other cargo to the plasma membrane (PM) and by exocytosis transit these vesicle contents to the extracellular matrix (ECM). Moreover, there is evidence that the ER/G contributes to the autophagic process [110-112] by donating proteins to phagophores (PH) [110] which, when enclosing a damaged organelle like a mitochondrion, form autophagic (mitophagic) vacuoles during auto(mito)phagy [116]. In some cases, such mitophagic vacuoles may contain calcium and phosphate ions, ion clusters and granules (Ca—P), which are ultimately liberated to the ECM [136]. mTOR (mammalian target of rapamycin complex) and AMPK (AMP kinase) coordinately regulate PH formation [113], and these organelles develop into autophagic vesicles, autophagosomes (APS) [108]. APS may fuse with lysosomes to form autophagolysosomes (APLS), which degrade their enclosed cargo [108]. Alternatively, APS coordinated with autophagy proteins (ATG) may fuse with early endosomes to form amphisomes [117-119]. These organelles traffic to the cell envelope, interact with the PM and move their contents to the ECM. Lysosomes may also fuse with late endosomes to generate endolysosomes (ENLS), which, like endosomes alone, harbor high levels of calcium ions but carry a spectrum of hydrolytic enzymes as well that cause cargo breakdown [108]. Undergoing exocytosis, ENLS deliver their contents to the cell exterior. TRPML1 (transient receptor potential mucolipin 1) [179,181] is the principal calcium ion release channel in the ENLS. Lysosome biogenesis and exocytosis are controlled by transcription factor EB. Early endosomes derived from membranes of the ER traffic calcium ions, proteins and additional molecules to the PM, as noted above, and other endosomes that originate at the PM can import similar cargo into the cell. Both early and mature (late) endosomes laden with cargo may transit the cell and form multivesicular bodies (MVBs) in the presence of ubiquitin and ESCRT (endosomal sorting complexes required for transport) proteins [26]. MVBs contain numerous smaller intraluminal vesicles (ILVs) also carrying cargo. Directed by SNAREs, Rab-GTPases, LC3 (microtubule-associated protein 1 A/1B light chain 3), and other proteins, fusion of the MVB with the PM occurs and subsequently the ILVs and their contents are released to the extracellular milieu as exosomes/matrix vesicles. Phosphate ions (Pi) in the form of ${\text{HPO}}_{4}^{2-}$ and $H_{2}{\text{PO}}_{4}^{-}$ appear in the ECM of cells and tissues, and these two species are present in intracellular organelles [26]. Pathways of phosphate ion cell entry and intracellular transit are not as well understood as those of calcium ions. Phosphate ions are documented in mitochondria and undoubtedly are ferried as cargo in early and late endosomes, ENLS, APLS, amphisomes, MVBs and ILVs [26]. These ions, like calcium ions, find themselves released to the ECM as free ions or associated with calcium ions in calcium-phosphate ion clusters or early mineral phases such as amorphous calcium phosphate. MAM denotes the mitochondria-associated endoplasmic reticulum membrane, a protein complex linking the ER and mitochondria for calcium ion transport as described earlier. (Figure adapted from Landis and Shapiro [26] with permission. Figure created using BioRender.com.)

Forty years ago, Reith showed that foci of calcium ions were evident in the endoplasmic reticulum during amelogenesis [77]. Not known at that time was the fate of these ions and the possibility that they might be released from the endoplasmic reticulum to the enamel extracellular matrix. While there is still much to be learned of the processing and trafficking of calcium, as well as phosphate, ions in enamel and other vertebrate hard tissues, considerable evidence now exists to indicate that calcium ions can be directly transferred into many different types of vesicles that originate or associate with the endoplasmic reticulum (Fig. 4). Alternatively, rather than direct transfer, these ions released into the cytosol may be accumulated by the numerous endosomes, endolysosomes and autophagosomes that populate the cells of the skeletal and dental tissues (Fig. 4). These organelles comprise in part the endolysosomal and autophagic systems of the cells and functionally are responsible for intracellular transport of their cargo, some of which may be exocytosed to the extracellular matrix. Specifics about these vesicular organelles and their transport systems are addressed later in this review.

The mechanism of ion release from the endoplasmic reticulum involves ligand binding to cognate receptors present in the reticulum membrane. The ligand is generated by the activity of the plasma membrane enzyme, phospholipase C, which hydrolyzes the phospholipid, phosphatidylinositol 4,5-bisphosphate, into the water-soluble inositol 1,4,5-trisphosphate (IP3) noted previously. When IP3 binds its cognate receptors, IP3R, the ligand-gated channel opens and discharges calcium ions into the cytosol, mitochondria (through the MAMs) or endolysosomes and other vesicles [78]. Confirming the latter observation, Garrity and colleagues showed that IP3 antagonists prevented calcium refilling of endolysosomes [79]. In addition to IP3, a second calcium-releasing ligand, cyclic ADP-ribose (cADPR), is present in cells, synthesized from NAD(P) by cADPR cyclase [80]. This ligand binds RyR in the membranes of the endoplasmic reticulum and releases calcium ions into the cytosol [80]. Paradoxically, cADPR also promotes the activity of SERCA [81]. From this perspective, ligand binding appears to provide a subtle and complex cue that influences both calcium ion loading and release from the endoplasmic reticulum.

There should be no surprise that IP3 regulates calcium release from the endoplasmic reticulum of vertebrate hard tissue cells. Shibukawa and Suzuki showed, for instance, that odontoblasts express phospholipase C-coupled receptors [82]. Activation of the receptors and the synthesis of IP3 caused the endoplasmic reticulum to discharge calcium ions [82]. A similar system was found to be active in epiphyseal growth-zone cells, where an increase in IP3 levels liberated calcium ions from the endoplasmic reticulum of chondrocytes [83]. It should also be acknowledged that there is some evidence that RyR may be active in osteoblasts [84]. These studies lend direct support to the concept that a common system exists for releasing calcium ions from subcellular stores.

As indicated earlier, besides their immediate release into the cytosol, calcium ions are directly transferred to the cytosol through endoplasmic reticulum vesicles such as endolysosomes and lysosomes [78]. The transfer process is facilitated by the shared presence of specialized proteins comprising the membrane of both organelles. For example, Pihan and Hetz reported that calcium ion transfer to lysosomes was mediated by the endoplasmic reticulum membrane protein, transmembrane BAX inhibitor motif containing 6 (TMBIM6) [85]. This channel protein sensitizes IP3R for its cognate ligand (IP3), an action resulting in a decrease in calcium ions in the endoplasmic reticulum and a corresponding increase in lysosomal calcium ion levels [86].

The influence of TMBIM6 is not confined to lysosomal loading of soft tissue cells alone as the protein is present at elevated levels in both osteoblasts and osteoclasts and its deletion causes development of an osteoporotic-like phenotype in *TMBIM6*-knockout mice [87]. Consistent with its impact on bone cells, TMBIM6 influences the mineralization status of dental tissues. The protein is secreted in ameloblasts and odontoblasts of the developing tooth germ [88]. In other *TMBIM6*-knockout mice, differentiation and maturation of both cell types are disturbed [88]. These findings reinforce the view that the endoplasmic reticulum serves as a source of calcium ions for endolysosomes and lysosomes [79]. In this regard, it would be safe to conclude that high levels of cytosolic calcium ions are initially dependent on their influx from the extracellular environment mediated by the SOCE system noted previously and by the subsequent transfer of these ions from stores in the endoplasmic reticulum to vesicles of the endolysosomal system. This latter topic concerning ion-laden intracellular vesicles will be elaborated in the coming paragraphs of this review.

As an additional consideration addressing calcium ion release from the endoplasmic reticulum, it should be mentioned that the ion is discharged from the organelle during protein translocation [16]. In this respect, the Sec61 translocon imports nascent polypeptides into the endoplasmic reticulum while maintaining the permeability barrier between the cytosol and the endoplasmic reticulum lumen [89]. During protein translocation, transient calcium ion efflux into the cytosol is mitigated by a gated “leak” channel that is regulated by a calcium-dependent molecular chaperone, immunoglobulin heavy chain binding protein, BiP [90,91]. These activities also involve several co-chaperones, including J-domain proteins and a nucleotide exchange factor [92].

To summarize, cytosolic calcium ion homeostasis is dependent on the activities of a complex series of plasma membrane and mitochondrial pumps and gates. Cells sustain low cytosolic calcium ion levels through controlled passage of these ions into the endoplasmic reticulum and other organelles. As a result, there is both direct cytosolic calcium ion loading of the endoplasmic reticulum and calcium ion loading of vesicles derived from or associated with the endoplasmic reticulum. Additionally, it is plausible to consider that ATP-derived phosphate ions liberated in reactions during protein synthesis provide the calcium counterion for mineral formation. In the very low volume vesicle environment, certain changes in Gibbs free energy could favor calcium and phosphate ion clustering and possible formation of prenucleation mineral species. Details of how vesicles transport such cargo are considered in the following sections of this paper.

## 4. Nature of the vesicular systems that may transport calcium phosphate cargo to the plasma membrane and extracellular matrix

In a rapidly mineralizing tissue such as endochondral bone, large quantities of ions and proteins such as collagen and additional matrix macromolecules must be transported to the cell exterior in a highly regulated manner. The first step in this process is the formation of numerous, different vesicles from the endoplasmic reticulum or the plasma membrane as noted above and the inclusion of their cargo of ions and proteins (Fig. 4). This step is followed by the acquisition of other proteins that bind to the external surfaces of vesicle membranes and act as molecular “zip codes” in sorting the various vesicles and targeting them to specific intracellular organelles or locations. Motor proteins (kinesins and dyneins) as well as structural cytoskeletal proteins (actins and myosins) help guide and shuttle the separate vesicles to their destinations. The last stage is docking and fusion of the vesicles with their intracellular target organelles or membranes. In this way, the vesicle cargo fated for secretion is parted from that needed for cellular housekeeping functions. In vertebrate mineralizing tissues, those vesicles containing calcium and phosphate ions, ion clusters or possible mineral phases destined for secretion are separated from those remaining within the cytosol. Necessary housekeeping can be maintained while there is simultaneous trafficking of key inorganic and organic components to the plasma membrane and extracellular milieu.

The exact types of vesicles that participate in intracellular calcium phosphate transport are incompletely understood. This statement will not come as a surprise to those readers who have observed the fine structure of living cells and who are cognizant of the bewildering complexity and density of transport organelles that populate the cytosol. Despite this uncertainty, some vesicles identified experimentally appear more likely than others to be involved with the intracellular transport of calcium and phosphate ions to the plasma membrane and ultimately to the extracellular matrix as critical mediators of the mineralization process in vertebrate hard tissues. The principal vesicles in this context, endosomes, lysosomes and autophagy-related vesicles, are considered below together with matrix vesicles.

Endosomes are common small membrane-enclosed vesicles formed by invagination of the plasma membrane or originating in the membranes of the endoplasmic reticulum of somatic cells (Fig. 4). The invagination process leads to internalization of cellular and extracellular constituents: such cargo may be membrane-related ligands, other macromolecules and inorganic ions [93,94]. Endosomes may also receive proteins synthesized in the endoplasmic reticulum [94]. The fate of endosome cargo is complex as some cargo may be directed to the plasma membrane, some may undergo lysosomal degradation, or some may be trafficked to the trans-Golgi network (retrograde transport) [94]. A means of intracellular cargo movement was investigated by Chakrabarti et al., who showed that a “dense filamentous cortical actin proximal to the plasma membrane provides a physical path for newly formed endosomes to traverse further into the cytoplasm.” [95] Newly formed (early) endosomes at the plasma membrane and endoplasmic reticulum are highly dynamic organelles, exhibiting morphologies that vary from large spherical structures (~60 nm diameter) to vesicles with tubular extensions (~400 nm diameter) [96]. While derivatives of the plasma membrane phospholipid, phosphatidylinositol, play a major role in organizing and trafficking vesicles and mediating membrane fusion, the GTPase binding protein, Rab5, among the many cellular regulatory proteins, controls early endosome morphology and function [94,97]. Replacement of Rab5 to Rab7 marks maturation of early endosomes to late endosomes and formation of a multivesicular body containing numerous intraluminal vesicles that are released from the cell as exosomes (Fig. 4) [97].

The maturing (late) endosome may form hybrid organelles with both autophagosomes (amphisomes) and lysosomes (endolysosomes) (Fig. 4), and it may also interact to create membrane contact sites (10–30 nm in length) with the endoplasmic reticulum [97,98]. Friedman and co-workers estimated that over 99% of all late endosomes develop the latter type of inter-organelle connections [98]. The late endosomes receive cargo from the trans-Golgi network, including transmembrane sorting receptors, such as mannose 6-phosphate receptors (MPRs) and sortilins (Vps10p domain receptor family), which serve to target endosomes to specific cellular locations [99-101].

Endosomes can obtain calcium and phosphate ions from at least two sources. Calcium and likely phosphate ions, both originating in the endoplasmic reticulum, are present in endosomes and multivesicular bodies (Fig. 4). Both ions may also be taken up from the extracellular tissue fluid during endocytosis. Additionally, these ions are released from mitochondria as has been discussed previously [102]. The calcium and phosphate ions contained in endosomes would be expected to be targeted by the transmembrane sorting receptors to specific intracellular locales, as mentioned above, and released by exocytosis from the cell into the extracellular milieu. From this viewpoint, the endosome system together with autophagosomes and lysosomes would act as a vehicle for the transport of calcium and phosphate ions to the mineralization front. Moreover, as discussed later, thermodynamic/physicochemical changes within these vesicles as they move intracellularly may facilitate the formation of ion clusters and possibly patent early mineral phases.

Lysosomes are variably sized enzyme-filled vesicles, present in large numbers in osteoblasts and other hard tissue cells [103] and derived from membranes of the endoplasmic reticulum (Fig. 4) [104]. Their biogenesis is complex and can be viewed as a “progressive maturation and acidification of vesicular intermediates along the endocytic pathway.” [105] The interior of the lysosome is acidic (pH 4.6–5.0), maintained by membrane proton ATPases (H^+^ATPases) which pump protons from the cytosol across the lysosomal membrane [104]. The prime function of lysosomes is to degrade cargo contained in endocytic and autophagic vesicles, utilizing a wide variety of acidic hydrolases [104]. Lysosomes are also active in membrane repair, gene regulation, metabolic signaling, immunity, and cell adhesion and migration [104,106]. As noted earlier, these vesicles contain elevated levels of calcium ions [107]. Fused with autophagosomes to form autophagolysosomes, these organelles are also involved in the efficient degradation of their internalized contents and may release their cargo to the cell exterior as do endolysosomes described above (Fig. 4) [85,108,109].

Autophagy-related vesicles include autophagosomes and other organelles that are critical to the process by which a cell scavenges, degrades, and recycles a host of intracellular material, thereby providing a means of its self-renewal [110-112]. Autophagosomes are derived from a cup-shaped structure, the phagophore, formed and controlled by mTOR (mammalian target of rapamycin complex) and AMPK (AMP kinase) [113] within a specialized autophagosome initiation complex that is likely derived from the endoplasmic reticulum membrane (Fig. 4) [114]. Following maturation of the phagophore, autophagosomes isolate damaged and spent organelles and cytosolic molecules, enveloping them in a double-membrane structure [111]. In this type of organelle, Rab7 promotes autophagosome fusion with lysosomes to form autophagolysosomes, which function to degrade and recycle their cargo as just noted [115]. Besides other cellular debris, this process removes impaired mitochondria from the cell (mitophagy), while preserving existing functional mitochondria and controlling the biogenesis of new organelles [116].

Commonly in the autophagic pathway, autophagosomes fuse with endolysosomes to form a hybrid vesicle, the amphisome, which, like the endolysosome, can traffic its contents intracellularly and liberate the cargo by exocytosis (Fig. 4) [108,109,117-119]. As will be discussed shortly, such cargo may contain high concentrations of calcium and phosphate ions in the cells of vertebrate mineralizing tissues.

On further consideration of vesicles that form the principal system for shuttling cargo from the cell to the plasma membrane, it is important to comment that direct connections exist between the endoplasmic reticulum and the plasma membrane as well as between the outer membrane of mitochondria and the plasma membrane. Indeed, Friedan and co-workers predicted that 10% of the plasma membrane is in contact with mitochondria [120]. The molecular structure of the endoplasmic reticulum-plasma membrane contacting surfaces is complex and includes several tethering proteins (Ice2p, Ist2p, Scs2p and Scs22p) and synaptotagmin-like proteins (Tcb1p-Tcb3p) [121,122]. Disruption of contact between the endoplasmic reticulum and plasma membrane by gene deletion causes dysregulation of phosphoinositide and calcium ion signaling [121,123]. Moreover, increased secretion of the endoplasmic reticulum protein, STIM1, even without changes in secretion of ORAI, leads to enlargement of inter-organelle contact areas [124]. The endoplasmic reticulum-plasma membrane contacting surfaces generate phospholipid inositides and calcium signaling molecules; these contacts can be rapidly disassembled to prevent cytotoxic calcium overload [123].

There is little question that contact sites between cellular components are important in terms of non-vesicular lipid transfer, potassium channel function and calcium homeostasis [125,126]. Currently, however, no information is available concerning the role of non-vesicular organelle platforms in osteoblasts or the cells of other vertebrate hard tissues as they relate to the mineralization process. Nevertheless, it is tempting to speculate that these endoplasmic reticulum-plasma membrane contacting surfaces may provide yet another system for shuttling calcium and phosphate ions from the cell to the extracellular matrix.

Matrix vesicles are another type of vesicular organelle that has been conventionally associated with mineralization of vertebrate tissues, more specifically endochondral bone and early intramembranous bone and dentin (Fig. 4). It should be noted that vertebrate mineralization may be mediated by not only matrix vesicles but also collagen and amelogenins, for example, but a discussion of these additional means of mineral deposition is not appropriate to vesicles in general and is therefore outside the scope of this review.

Unlike endosomes or any of the other vesicles discussed earlier, matrix vesicles are extracellular membrane-invested structures, ~50–100 nM in diameter, frequently located near or associated with extracellular sites of mineralization in many collagen-based hard tissues [127-129]. Indeed, at such sites, matrix vesicles have been reported to initiate extracellular mineral formation and/or mediate mineral deposition within collagenous matrices [127,128].

Early electron microscopic studies indicated that matrix vesicles were formed by polarized budding or blebbing from plasma membrane protrusions [127,130,131]. However, more recent investigations suggest that other mechanisms of vesiculation exist. Thus, Akisaka et al. reported that some vesicles appeared to pass through intact plasma membranes [132], while Xiao et al. noted that osteoblast vesicle biogenesis was mediated by the budding of aggregate clusters of vesicles contained in membranous structures originating at the cell surface [133]. Additionally, Thyberg et al. regarded these vesicles as extrusions of lysosomal dense bodies [134]. Based on these results, it would seem that several mechanisms may give rise to matrix vesicles (Fig. 4).

Matrix vesicles are reported to contain more than 280 proteins, including TNAP, annexins, phospho-1, nucleotide triphosphate pyrophosphatase, and phosphodiesterase as well as extracellular matrix metalloproteases [129,133]. Integrin receptors in the matrix vesicle membrane mediate interactions with collagens, fibronectin, and laminin, as well as cell adhesion molecules [129]. These interactions are critical to the formation of extracellular mineral mediated by matrix vesicles as noted below.

## 5. Identification of calcium phosphate cargo carried by intracellular and extracellular vesicles

Evidence is compelling that vesicles associated with the endoplasmic reticulum and mitochondria transport mineral cargo (Figs. 2, 4). Tang et al. observed electron-dense granules of ~10 nm in diameter located around the endoplasmic reticulum, in addition to those present in vacuolated mitochondria [135]. Morphologic changes suggested that mitochondria containing biomineral precursors were undergoing mitophagy [135]. That vesicles and the autophagic pathway were involved in transporting mineral was further investigated by Nollet and co-workers using mutant mice that exhibited vesicular deficits [136]. This group demonstrated that autophagic vacuoles contained mineral and inhibition of the autophagic response prevented osteoblast function. Additionally, the phenotype of the autophagy-mutant mice was characterized by a 50% reduction in trabecular bone mass. In this instance, a conditional knockout of the autophagic gene, *ATG7*, blocked osteoblast mineral deposition in the animals but absence of the block restored mineral formation [136]. In a subsequent investigation, Li et al. confirmed the importance of autophagy in mineralization. These investigators inhibited autophagy and reported a loss of activity of the mineralization-associated enzyme, TNAP [137]. Further study, however, indicated that manipulation of the autophagic pathway could be used to promote mineralization. In this case, Behera and colleagues found that autophagy was stimulated and bone mineralization was upregulated in cells treated with autophagy-inductive silica nanoparticles [138].

The nature of the cargo carried by intracellular vesicles in osteoblasts was examined by Boonrungsiman et al. using a cell culture model of bone formation [139]. Analytical electron microscopy identified calcium phosphate particles both within intracellular vesicles and mitochondrial granules [139]. Many of these calcium-containing vesicles were adjacent to mitochondria, a result suggesting that the mitochondria were experiencing mitophagy, as subsequently noted by Tang et al. [135] mentioned above. Selected area electron diffraction showed that the vesicle cargo was predominantly an amorphous calcium phosphate, and the data indicated that, if crystalline apatite or other intermediate mineral phases were also present in the vesicles, those mineral species would represent only a few unit cells [139].

In another investigation, cryo-electron microscopy was utilized to examine the mineral content of cells in frozen-hydrated mouse calvaria and long bones [140]. In this work, it was reported that bone-lining cells concentrated membrane-bound mineral granules in intracellular vesicles during mineral formation [140]. While the nature of the vesicles was not determined, the mineral was considered to be an amorphous calcium phosphate [140]. The calcium content of the granules was low, a result that suggested a phosphate-rich mineral may have first formed and then transitioned into a more conventional amorphous mineral phase consisting of 80 nm diameter particles [140]. Within each 80 nm particle, smaller subunits, possibly representing Posner particles [Ca_9_(PO_4_)_6_], were present [140].

An additional study conducted by Iwayama et al., utilizing scanning electron-assisted dielectric microscopy to image live cell cultures of murine osteoblasts, showed that their intracellular vesicles identified as lysosomes also contained amorphous calcium phosphate [141]. To determine whether the lysosomes were involved with trafficking the mineral, the cells were treated with bafilomycin, an inhibitor of lysosomal H^+^ATPase. Under this condition, there was a loss of acidification and a marked reduction in electron dense calcium-stained granules in the lysosomes [141]. Moreover, the lysosomes remained restricted to the cytosol, an observation suggesting that the mineral cargo was released from the cells by exocytosis. When exocytosis was inhibited with vacuolin, lysosomes with intact stained mineral were found confined to the cell [141]. These findings provided powerful evidence that lysosomes and possibly fusion vesicles such as autophagolysosomes are involved with intracellular mineral ion uptake and the formation and transport of early mineral phases of calcium phosphate.

Regarding matrix vesicles, numerous reports using electron microscopy and other methods appear to demonstrate the presence of ions, ion clusters and/or mineral phases within these organelles [127,128,142,143]. The ions or mineral are formed in association with the matrix vesicle membrane or the vesicle internal compartment after the vesicle has been released from a cell to become attached firmly to the extracellular matrix through its membrane receptors as noted previously [129]. A calcium-phosphate nucleating complex has been proposed to facilitate the putative mineral nucleation events of matrix vesicles [144].

## 6. Sorting of vesicular organelles associated with mineralization, their transport and interaction with the plasma membrane

As mentioned earlier, a microscopic view of a cell in real time shows a highly complex arrangement of constituents in which the intracellular vesicles and other structures of the cytoplasm and nucleoplasm are in constant motion and a state of apparent chaos. Of course, this is not at all the case as these ostensibly random movements hide underlying well defined and strictly controlled patterns, interconnections, feedback loops and circuits of the jostling cellular molecules and organelles. How coordination of these activities is achieved and how a cell organizes and prioritizes its seemingly infinite number of functions has yet to be determined. This complexity of function is especially relevant to the mineralization process, where a cell must deposit mineral in its extracellular matrix while elaborating the proteins, proteoglycans and other components of the organic matrix. This duality of purpose raises the following questions: how are the many intracellular vesicles laden with mineral ions, ion clusters, and possible mineral phases, as described above, distinguished, separated and sorted from those other vesicles concerned with fulfilling biosynthetic and housekeeping functions; how do the types of vesicles carrying mineral ions or mineral-related cargo move within the cytoplasm to the plasma membrane; what mechanism(s) governs the interaction and fusion of these vesicles with the cell envelope; and how are the vesicles or their contents liberated to the extracellular matrix? These questions are addressed in subsequent paragraphs of this section of the review.

**Fig. 5.**
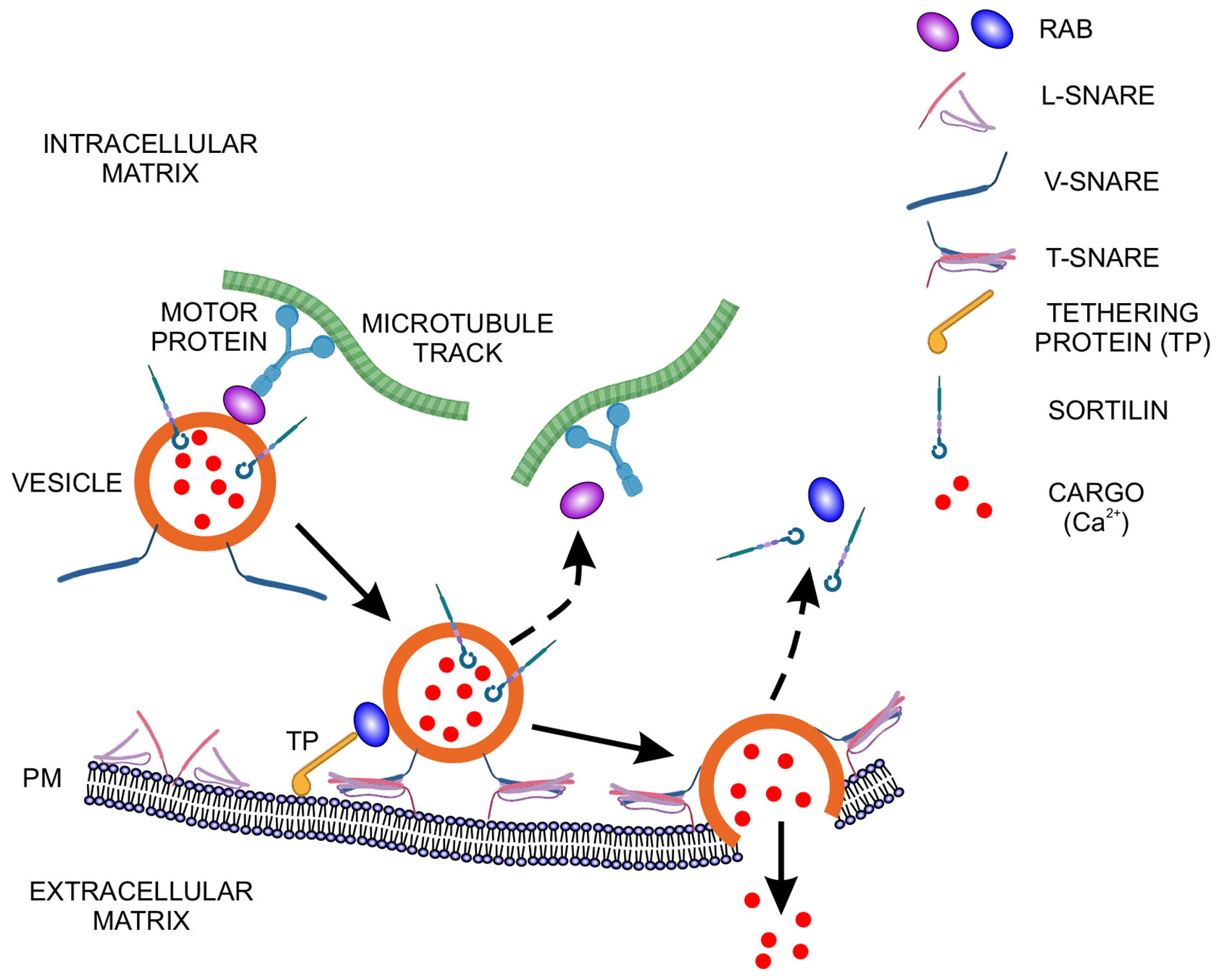
Schematic of cargo trafficking within eukaryotic cells utilizing vesicle carriers originating from the endomembrane system of the cell. Fusion of transported vesicles at their destination membrane requires the coordinated action of intracellular Rab-GTPases, tethering complexes, SNAREs and other molecules. Vesicles derived from the Golgi bind sortilins which serve as sorting receptors for cargo intended for transport to the plasma membrane (PM) and/or the lysosomal pathway [152,155]. These vesicles are recognized and bound by Rab proteins and motor proteins such as kinesins and transported along cytoskeletal (microtubule) tracks [145,146]. During transport, particular V (vesicle)-SNARE proteins also bind to the surface of vesicles, conferring specificity for membrane docking. Other Rab-GTPases together with tethering proteins (TPs) recognize vesicles and draw them closer to the plasma membrane. The subsequent docking of vesicles on the membrane is then mediated by interaction of Rabs, TPs, and a complex of V-SNAREs with complementary L (loose)-SNAREs [146]. The association of the V- and L-SNAREs (as so-called T (target)-SNAREs) promotes fusion of the vesicle and plasma membranes and cargo release [146]. Phosphate ions, calcium-phosphate ion clusters or possible early mineral phases may also be carried by vesicles as cargo as described above. Dashed arrows indicate dissociation of different cellular components from vesicles and recycling of sortilins and Rab proteins. (Figure created using BioRender.com.)

The question posed earlier concerned mechanisms for separating and sorting mineral ion-containing vesicles from those concerned with housekeeping and secretory activities has received limited study. In contrast, a great deal is known of the sorting and vesicular fate of many soluble proteins synthesized at the endoplasmic reticulum/Golgi complex. This sorting function is mediated by the attachment of specific Golgi proteins to the outer membrane of the cargo-containing vesicles, and such proteins help direct the organelles to a particular intracellular or plasma membrane destination, the molecular “zip coding” referred to previously in this paper. For example, soluble hydrolase enzymes that exhibit mannose-6-phosphate residues bind to cognate receptors in clathrin-coated vesicles formed from Golgi membranes [101]. As these and other vesicles are moved along the tracks of microtubules by kinesin motors (Fig. 5) [145], their cargo interacts with the inner aspect of the vesicle membrane through cargo adaptor proteins that target the vesicle to endosomes/lysosomes, the Golgi complex, the plasma membrane, or elsewhere [101]. Following such sorting, endolysosomes acquire SNARE (soluble N-ethylmaleimide-sensitive factor attachment protein receptor) and Rab-GTPase proteins which enable their trafficking to specific cellular compartments or locations where they then associate with various cell constituents, or the plasma membrane as just noted (Fig. 5) [101,146]. For endocytic vesicles originating at the plasma membrane and away from the Golgi, early-stage endosomes may be sorted along three different pathways, one that recycles back to the cell surface, one that progresses to late-stage endosomes, and one that generates lysosomes [147]. Early- and late-stage endosomes may be sorted through the action of ESCRT (endosomal sorting complex required for transport) proteins and transitioned into multivesicular bodies, which themselves may form intraluminal vesicles (Fig. 4) [26,148,149].

Although mannose-6-phosphate receptors play a major role in the intracellular transport of newly synthesized endolysosomal enzymes, there is some evidence in vertebrate mineralizing tissues that endocytic sorting is also dependent on the presence of other transmembrane receptors, sortilins (vacuolar protein sorting 10 proteins, Vps10p) and the sortilin-related receptor, SorLA (Fig. 5) [150]. The sortilin protein, elaborated in the Golgi complex [99], is a 10-bladed β-propeller-like structure with a coiled-coil region [150]. The molecular structure of the Vps10p propeller promotes binding to more than 50 different ligands [150]. The expression of sortilin (*SORT1*) is tightly regulated at the transcriptional, post-transcriptional, and post-translational levels in a cell- and tissue-specific manner [151]. If *SORT1* is deleted, vesicle cargo becomes trapped and its delivery to lysosomes is impaired [151]. Sortilin recognizes and binds early/late-stage endosomes and lysosomes and targets these vesicles to intra- or extracellular destinations [100,152].

*Sortilin* was initially reported to be expressed by mesenchymal stem cells undergoing osteoblastic differentiation and its overexpression promoted tissue mineralization [153]. In subsequent studies, Boggild et al. noted that sortilin was detected at numerous sites of mineralization, including those in tissues undergoing intramembranous ossification [154]; and Goettsch and colleagues reported that sortilin in the mineralization of vascular tissues facilitated the loading, phosphorylation and transport of TNAP, a critical component of the mineralization tool kit [155]. Contained in Rab11-positive endosomes, TNAP was trafficked from the trans-Golgi network to caveolin-enriched domains of the plasma membrane [155]. Goettsch et al. also reported that patients with chronic renal disease secreted sortilin in the tunica media and that its expression was high in human calcified atheromatous plaque [155]. How this and related proteins are involved in mineralization of the dental tissues has yet to be established.

## 7. Vesicular transport

As mentioned previously, the trafficking of vesicles, vesicle cargo and protein complexes to specific intracellular locales is conducted primarily along the microtubule network of the cell and the actin cytoskeleton, powered in part by the motor proteins, kinesin, dynein and myosin [145,156,157]. To accomplish such movements, the kinesin and dynein family of motor proteins move cargo on microtubule tracks or pathways, while the myosin family of motors moves organelles along the branched complex principally consisting of the filamentous protein, actin [157]. In both cases, ATP hydrolysis provides the chemical energy for such actions, and cytoskeletal activity is mediated by a wide range of actin–microtubule crosslinking proteins [158,159]. Actin and microtubules do not have clearly distinct functions but rather participate together in the majority of transport functions [160,161]. According to Hohmann and Dehghani, actin is the most dynamic of the cytoskeletal proteins [162]. The branched actin complex is formed by a member of the Wiskott-Aldrich syndrome protein (WASP) family activated by an actin-related protein 2/3 complex (Arp2/3) [163], and this catalyzed cytoskeletal component directs the outward flow of vesicles from the endoplasmic reticulum toward their interaction with one another or their docking at the plasma membrane [164].

While the actin network provides a system for the outward transport of vesicles from the endoplasmic reticulum, the volume and diversity of various cargo requires that trafficking be tightly regulated. As noted above, the intracellular routes of vesicles are further defined by members of the large family of Rab-GTPases together with additional Rab proteins, tethers, SNAREs, and yet other protein species [146,147,164,165]. Here, specific Rab-GTPases recruit motor proteins, sorting adaptors and tethering factors that critically guide vesicles to their correct destination and such Rab proteins also assist in vesicle docking with the plasma membrane [164,166]. Moreover, Rab proteins control fusion between intracellular vesicles and between vesicles and the plasma membrane [26].

Although vesicle transport is better understood, information on the mechanism(s) by which calcium and phosphate ions, initially accumulated in the endoplasmic reticulum or mitophagic vacuoles, are transported to the plasma membrane is less studied. As described earlier, mineral ions or their clusters present in the endoplasmic reticulum membrane are incorporated into forming endosomes or endolysosomes as vesicle cargo. All of these several organelles, including multivesicular bodies, may transit their cargo to the plasma membrane and export the contents to the extracellular milieu (Fig. 4) [26].

In contrast to endosomes, autophagosomes transport cargo to the plasma membrane and release proteins, lipids, ions and other molecules to the cell exterior. This form of secretory autophagy is dependent on autophagy (ATG) proteins, Golgi reassembly stacking protein (GRASP), Rab8a and ESCRT proteins, while SNAREs promote autophagosome fusion with the plasma membrane (Fig. 4) [118,164,165]. Moreover, there is evidence that the endoplasmic reticulum contributes to the autophagic process [110,115,118], possibly by mTOR and AMPK coordinately regulating phagophore formation, as noted earlier in this review [113]. Rogov et al. pointed out that autophagosome formation, crosstalk with the endocytic network and autophagosome fusion with lysosomes are all interactions mediated by ATG8/9 binding to Unc-51-like autophagy activating kinases (ULK1/2) [167].

With respect to damaged or non-functional mitochondria, transcellular trafficking of mitophagic cargo occurs through sequestration of the debris in mitophagic vacuoles or vesicles [168]. These latter organelles can be degraded following fusion with lysosomes and distributed to other vesicles such as multivesicular bodies or amphisomes [108,169]. Fregno and Molinari reported that fragments of the endoplasmic reticulum membrane itself may be taken up by autophagosomes, a process called ER-phagy [170].

It should be noted that currently only one study has been performed to evaluate the expression of vesicle trafficking genes during osteogenesis. Zhu et al. silenced vesicle trafficking genes *Gosr* (*Golgi SNAP receptor complex member*)*2, Arf* (*ADP ribosylation factor*)*4, Cog* (*conserved oligomeric Golgi complex*)*6*, and *Pacs* (*phosphofurin acidic cluster sorting protein*)*1* and found RNA depletion increased mineralized nodule formation [171]. Of these transcripts, *Gosr2* belongs to a group of SNAREs that are essential for budding, docking and fusion of COPII-coated vesicles during endoplasmic reticulum-Golgi membrane trafficking [171]. Moreover, permanent knockdown of *Cog6* and *Pacs1* by CRISPR/Cas9 gene editing promoted mineralized nodule formation and osteoblast differentiation [171]. These transcriptomic analyses clearly showed that vesicle components are required for mineralization. Zhu et al. hypothesized that the increase in mineralized nodule formation with knockdown of trafficking genes may be related to an increase in prolyl 4-hydroxylation (P4ha) of collagen that they found was linked to an elevation in *P4ha1* and/or *P4ha2* expression [171]. These investigators also commented that enhanced hydroxylation of collagen would be expected to “increase the space available for mineral deposition among the extracellular matrix.” [171] Alternatively, the deletion of many of the genes involved with vesicle function may disturb vesicle transport and coordinated organic-inorganic interactions, a result that leads to extracellular matrix hypermineralization.

These several studies presented above support the concept that the endosomal, lysosomal and autophagic pathways of a cell provide several different avenues for the intracellular transport of mineral ions, ion clusters or possibly early mineral phases. The observations strengthen the idea that the initiation of vertebrate mineralization occurs within the intracellular, rather than extracellular, compartments of a hard tissue.

## 8. Vesicle interaction with the plasma membrane and secretion to the extracellular milieu

The deposition of mineral in the extracellular milieu of vertebrate hard tissues is mediated by the export of the inorganic ion-containing cargo of intracellular vesicles described previously. However, the plasma membrane, while exquisitely regulating cell entry of these and other ions along with a multitude of molecules and additional biological species, as noted earlier, also serves as a physical barrier preventing liberation of ions and molecules from the cell. In this situation, several mechanisms may direct the manner by which cargo-laden vesicles are liberated from the cell. For example, lysosomes originating from the endoplasmic reticulum as well as vesicles developed through mitophagy may gain access to the cell exterior by docking and fusion with the cell membrane, processes that are themselves calcium-dependent [172,173].

Other intracellular organelles that may be released directly from the plasma membrane include exosomes originating from intraluminal vesicles of multivesicular bodies (Fig. 4) [174]. Relevant to this observation, Xiao et al. have shown that sacs of vesicles not unlike multivesicular bodies are shed from osteoblast-like cells in culture [175]. Fusion of vesicles with the plasma membrane may also lead to release of their cargo from a cell as free ions, ion clusters, or possibly early mineral phases. Thus, the cell employs a number of different approaches to release either intact cargo-bearing vesicles or the cargo alone to its extracellular surroundings.

There is limited study of the docking process in vertebrate mineralizing tissues. However, based on work with non-mineralizing specimens, docking events have been found to involve specific Rab-GTPases, SNAREs, motor proteins, sorting adaptors and tethering factors, as mentioned previously (Fig. 5) [146,147,164,165]. Rab-GTPases are located on the cytosolic face of vesicles and they direct interaction of these organelles with lipid rafts, specialized cholesterol- and sphingolipid-rich regions occurring on the inner leaflet and cytosolic face of the plasma membrane [176]. Tethering proteins maintain vesicle-raft associations while other proteins, flippases, floppases and scramblases, may modulate ATP- and calcium ion-dependent transport of phospholipids between inner and outer leaflets of the plasma membrane [176,177]. Changes in plasma membrane structure induced by these three translocases, combined with SNARE activity, enhance blebbing of the plasma membrane and liberation of intact cargo-containing vesicles to the extracellular milieu (Fig. 4) [174].

The discharge of mineral ion cargo from vesicles at the plasma membrane appears to involve two protein families: two-pore channels (TPC) and transient receptor potential mucolipin (TRPML) [178-182]. TPC proteins have been suggested to respond to nicotinic acid adenine dinucleotide phosphate (NAADP) [183,184] but are more likely activated by the phospholipid, phosphatidylinositol 3,5-bisphosphate (PtdIns(3,5)P_2_) [178,180]. Yamaguchi and collaborators have defined TRPML1-mediated exocytosis as a multi-step process dependent on activation of transcription factor EB (TFEB) [179]. TFEB triggers fusion of the vesicular membrane with the plasma membrane [172,179,185].

Aoki et al. showed that a high local concentration of sub-plasma levels of calcium ions delivered directly from the endoplasmic reticulum or indirectly from endoplasmic reticulum-associated vesicles causes a change in cytosolic fluidity [186]. This perturbation also elicits bleb formation at the plasma membrane [186]. The same group has shown that membrane blebbing involves ORAI1-STIM signaling as well [187,188]. Other factors influencing membrane structure and blebbing include a change in the balance of activities between two small GTPases, Rnd3 and RhoA, and the resulting dissociation of the plasma membrane from its underlying cytoskeletal network [187,188]. In a third possibility, autophagic vacuoles containing mineral particles would be expected to interact with the plasma membrane and cause a blebbing response [187]. This phenomenon would be attributable to release of the plasma membrane from the actin cytoskeleton. Myosins then associate with actin filaments, and actomyosin contraction would drive bleb retraction [189]. Delivery and exocytosis of the contents of the vacuoles again may provide a mechanism for the release of mineral cargo into the extracellular matrix. Plasma membrane blebs containing a cargo of ions, ion clusters or putative early mineral phase species would be released from the cell as intact membrane (matrix) vesicles (Fig. 4).

Having entered the extracellular matrix of the vertebrate hard tissues, the vesicles or their cargo become subject to further events leading to the onset and progression of mineralization outside the cell. The transit of vesicles, many of which contained mineral deposits, has been recently documented from their intracellular to extracellular locations in the femurs from developing chick embryos [190]. In this study, focused ion beam-scanning electron microscopy under cryogenic conditions that minimized specimen and technical artifacts was used to examine osteoblasts and/or preosteocytes and osteocytes [190]. By analyzing the mean volume of the vesicles containing mineral (0.65 μm^3^), the number of such vesicles per unit volume, and specific transport parameters, it was determined that the intracellular movement of the vesicles was not regulated by passive diffusion but rather involved an active transport process that provided much of the calcium required for tissue mineralization [190]. Delivery of calcium ions or mineral precursors shed from vesicles released from cells was suggested to be through an extensive three-dimensional nanochannel network penetrating the extracellular matrix of the femoral bone. Such a complex of nanochannels (~30–40 nm in diameter) has been described in avian leg tendon [191], the murine femoral growth plate [192], and human femoral cortical bone [193]. Thus, the extracellular matrices of vertebrate hard tissues become accessible to ion clusters as well as small molecules that contribute to mineral deposition of these tissue domains.

To conclude this section of the review, two principal intracellular structural networks have been identified that function to transport vesicles and their inorganic and organic cargo to docking sites and fusion with the plasma membrane. Following interaction between the vesicles and plasma membrane, these organelles and/or their contents are exported or secreted to the extracellular matrix where they may mediate mineralization of the milieu. Intracellular transport is associated with endosomes and autophagosomes, together with hybrid vesicles formed with lysosomes. Endosomes receive calcium ions directly from the endoplasmic reticulum or from the cytosol. As the ions may be concentrated in the closed compartments of endosomes, endolysosomes, multivesicular bodies, autophagic vesicles or their related organelles, free energy changes within these cellular components would promote ion clustering or possibly early mineral phase formation in the presence of phosphate ions presumed to be carried in the vesicles as well. At the same time, such invested vesicles would insulate the cytosol from abnormal shifts in calcium ion flux and its potentially damaging changes to sensitive cellular enzymic and signaling systems.

## 9. Summary consideration of the role of the endoplasmic reticulum and the endolysosomal and autophagic transport systems in vertebrate tissue mineralization

Based on the broad extent of information presented above, the central role of the endoplasmic reticulum and associated intracellular organelles in mediating the extracellular mineralization of vertebrate hard tissues may be summarized in the following paragraphs of this review. The possible importance of these organelles in this process is informed by observations that calcium ions are accumulated in the tubules and lacunae of the endoplasmic reticulum and Golgi complex. It is known that this process is mediated by plasma membrane pumps of which the SOCE system regulates the cytosolic calcium ion refilling response, while other membrane pumps promote the sequestration of ions in the endoplasmic reticulum/Golgi complex. In skeletal and dental tissues, gene knockout and silencing reports confirm that calcium ion uptake is dependent on SOCE function and that the endoplasmic reticulum/Golgi complex and mitochondria serve as major cellular reservoirs of calcium ions. Other studies indicate that high levels of calcium ions are present in vesicles derived from the endoplasmic reticulum and the autophagic transport system. With respect to phosphate, there are limited publications concerning its ion concentration in intracellular vesicles, but phosphate ion levels are likely high, like that of calcium ions. Phosphate ions that may originate in the endoplasmic reticulum could have their source from the putative elevated rates of release of inorganic phosphate ions from ATP during protein synthesis, folding and trafficking reactions in a cell.

Within the confined, hydrated environment of vesicles developed from the endoplasmic reticulum and the autophagic system, electrostatic interactions between sequestered calcium and phosphate ions could certainly be expected to form clusters. At a critical ion concentration and size in the crowded environment of the vesicle, some clusters may overcome the Gibbs free energy barrier to dissolution and, in doing so, lead to the formation of stable aggregates, putative prenucleation clusters, or very early phases of a calcium phosphate mineral species such as an amorphous calcium phosphate. Depending on the conditions within the vesicles, the latter cargo may undergo further thermodynamic or physicochemical transitions to generate mineral species that are of intermediate and progressively greater stability.

Besides the endoplasmic reticulum/Golgi complex and the autophagic pathway, it is likely that several key mineralization-related events depend on the activity of mitochondria. In its intimate relationship with the endoplasmic reticulum, this organelle maintains calcium homeostasis in the cytosol. Additionally, mitochondria are an important source of ATP, which may provide phosphate ions for mineral formation as noted earlier. Mitochondrial accumulations of calcium and phosphate ions are frequently seen as dense granules that may be trafficked to the plasma membrane if they are enclosed in autophagic (mitophagic) vesicles and vacuoles.

It is to be noted that, while the experimental support for a great majority of results presented in this review is robust, the findings concerning intracellular calcium homeostasis were principally made using cells of excitable and non-excitable soft tissues. In both cases, the focus of the investigations was to relate calcium handling to the regulation of activities that included cell cycling, apoptosis, neuroendocrine secretion and intracellular communication. Importantly, the cell calcium burden in these studies was low when compared with calcium levels required to bring about mineralization of skeletal and dental tissues. How cells of the hard tissues perform their housekeeping and biosynthetic functions while transporting the very large volumes of calcium and other ions required for mineralization of their extracellular matrices is largely unexplored and far from clear. Future work needs to address this issue so that the unique cellular events that lead to the deposition of mineral in vertebrates can be more fully understood.

This review has addressed the overarching question of the origin of mineral formation in vertebrate hard tissues, that is, whether mineralization of the extracellular matrices of skeletal and dental tissues is directed and dictated by intracellular or extracellular events. In this regard, extracellular matrix vesicles have been considered by many investigators to play a major role in mediating the mineral deposition outside the cells of bone, cartilage, dentin, cementum and the mineralizing tendons of some avian species. The present report, however, based in part on more recent developments in molecular and cell biology as well as high resolution microscopic techniques that limit artifacts in sample preparation, documents clearly that mineral ions, ion clusters and early mineral phases may be found within intracellular vesicles of endolysosomal and autophagic (mitophagic) transport systems. These vesicles transit the cell and, following their exocytosis or the release of their cargo, lead to the deposition of ions and/or mineral in the extracellular milieu. It may be concluded, then, that mineralization is initiated in the cell rather than through mineral ion-laden vesicles located in the extracellular matrix. Indeed, the trafficking of vesicles of endolysosomal and autophagic origin gives rise to matrix vesicles at the plasma membrane of vertebrate hard tissues where they may be liberated extracellularly. As such, this intracellular mechanism of endolysosomal and autophagic transport provides an exquisite level of cellular control of mineral formation, responsive to endocrinal and other factors, while providing cellular pathways for the movement of ions from the afferent blood supply to the mineralization front of vertebrate skeletal and dental tissues.

## Data Availability

No data were used for the research described in the article.
